# Fundamental Limits of Non-Orthogonal Multiple Access (NOMA) for the Massive Gaussian Broadcast Channel in Finite Block-Length

**DOI:** 10.3390/s21030715

**Published:** 2021-01-21

**Authors:** Jean-Marie Gorce, Philippe Mary, Dadja Anade, Jean-Marc Kélif

**Affiliations:** 1 Laboratoire CITI, Joint Laboratory between INRIA, The Université de Lyon and the Institut National de Sciences Appliquées (INSA) de Lyon, 6 Av. des Arts, 69621 Villeurbanne, France; dadja.anade-akpo@inria.fr; 2 Institut d’Electronique et des Technologies du Numérique (IETR) and Institut National des Sciences Appliquées (INSA) de Rennes, 20 Avenue des Buttes de Coësmes, CS 70839, 35708 Rennes, France; philippe.mary@insa-rennes.fr; 3 Orange Labs, 44 Avenue de la République, 92320 Châtillon, France; jeanmarc.kelif@orange.com

**Keywords:** superposition coding, many-user Gaussian broadcast channel, non-orthogonal multiple access, massive access, finite block-length, information theory

## Abstract

Superposition coding (SC) has been known to be capacity-achieving for the Gaussian memoryless broadcast channel for more than 30 years. However, SC regained interest in the context of non-orthogonal multiple access (NOMA) in 5G. From an information theory point of view, SC is capacity-achieving in the broadcast Gaussian channel, even when the number of users tends to infinity. However, using SC has two drawbacks: the decoder complexity increases drastically with the number of simultaneous receivers, and the latency is unbounded since SC is optimal only in the asymptotic regime. To evaluate these effects quantitatively in terms of fundamental limits, we introduce a finite time transmission constraint imposed at the base station, and we evaluate fundamental trade-offs between the maximal number of superposed users, the coding block-length and the block error probability. The energy efficiency loss due to these constraints is evaluated analytically and by simulation. Orthogonal sharing appears to outperform SC for hard delay constraints (equivalent to short block-length) and in low spectral efficiency regime (below one bit per channel use). These results are obtained by the association of stochastic geometry and finite block-length information theory.

## 1. Introduction

The Internet of things (IoT), connecting objects instead of humans, is one of the major applications of 5G and future generations of communications systems. Moreover, the transition towards machine to machine communications induces an important shift from the theoretical modeling of these systems. Indeed, the IoT paradigm relies on bursty but massive distributed communications to comply with the transmission requests of billions of communicating objects spread over a large area, while transmitting only few packets per day, month or even per year. In such a scenario, the classical fundamental limits of communication systems derived using the tools introduced by Claude E. Shannon [1] need to be revised. From this perspective the capacity, or the capacity region in case of multi-user communications, becomes less important in regard to other metrics [2,3].

The seminal Shannon’s second theorem established the capacity in additive white Gaussian noise (AWGN) channel, which can also be expressed as the fundamental trade-off between energy efficiency (EE) $\eta_{E}$ and spectral efficiency (SE) $\eta_{S}$ [4]. Let us define $\eta_{S}:$ = R/W where *R* and *W* are, respectively, the rate in bits/s and the channel bandwidth in Hz, respectively. In an ideal system, the number of channel uses (symbols in a narrow-band transmission) is 1/W and $\eta_{S}$ can be expressed in bits per channel use (bpcu). Now, letting the energy *E* be normalized with respect to the noise power density $N_{0}$, the energy efficiency is defined as $\eta_{E}:\;=\frac{RT}{E/N_{0}}$, where *T* corresponds to the transmission duration. By using the definition of $\eta_{S}$, the following relation holds $\eta_{E}=\eta_{S}\frac{\sigma^{2}}{P}$, with the average power P=E/T and $\sigma^{2}=WN_{0}$. Therefore, the energy efficiency can be alternatively thought as a power efficiency metric. In the following, by a slight abuse of notation, we will give to $\eta_{E}$ the dimension of bits per relative power unit (bppu).

Using this notation, Shannon’s channel coding theorem can be written as follows:

(1) $$\eta_{E}\leq \frac{\eta_{S}}{\left(2^{2\eta_{S}}-1\right)}.$$

This trade-off is achievable only in the asymptotic regime, i.e., when the encoding time spreads over an infinite number of channel uses (c.u.).

This asymptotic result relies on two assumptions which are no longer valid in the context of the IoT paradigm:

- the traffic is characterized by a continuous data flow;
- the encoding length is over an infinite number of channel uses and, hence, without any latency constraint.

While modeling IoT packets consisting of a few information bits under ultralow latency constraint (ULLC), the asymptotic regime becomes irrelevant. The first attempts to derive fundamental limits in the non-asymptotic regime dates back to Feinstein and Shannon in the 1950’s [5,6]. They provided an achievability bound on the rate considering maximal and average decoding error probability, respectively. A refinement on these results including cost constraints on codewords has been provided by Gallager [7]. The problem of achievability and converse bounds on the rate in the finite block-length (FBL) regime has recently received a renewed interest with the work of Polyanskiy et al. [8], who studied the fundamental limits of the point-to-point AWGN channel. This has paved the way to study latency and reliability constraints from a fundamental point of view. One of the major results in [8] is the asymptotic expansion of the achievable rate *R*

(2) $$R=C\left(\gamma \right)-\sqrt{\frac{V\left(\gamma \right)}{n}}Q^{-1}\left(\epsilon \right)+O\left(\frac{\log n}{n}\right),$$

where $C\left(\gamma \right)=\frac{1}{2}\log_{2}\left(1+\gamma \right)$ is the channel capacity in bpcu per dimension under the signal-to-noise ratio γ, $V\left(\gamma \right)=\frac{\gamma (\gamma +2)}{2{\left(\gamma +1\right)}^{2}}{\left(\log_{2}e\right)}^{2}$ is the channel dispersion defined as the variance of the information density between emitted and received codewords, *n* the number of c.u., ϵ being the error probability (average or maximal), and $Q\left(x\right)=1/\sqrt{2\pi }\int_{x}^{\infty }\exp\left(-y^{2}/2\right)dy$. This work has been extended to the multi-antenna case and fading channels in [9,10,11].

The work initiated in [8] has an impact far beyond theory and is of great interest for practical specifications for IoT networks, since the expression in Equation (2) links three fundamental constraints, identified as critical for IoT; i.e., reliability, latency or spectral efficiency, and energy efficiency [12].

### 1.1. Multi-User Finite Block-Length: State of the Art

Information theory has been proved to be a powerful tool to establish fundamental limits of point-to-point (P2P) or multi-user communication systems including the multiple access channel (MAC) and the broadcast channel (BC). These models fit well with the uplink and the downlink in a wireless cell, respectively. In the asymptotic regime, the exact characterization of achievable rate regions has been obtained [2].

According to [13], Gaussian MAC and BC capacity regions are dual of each other, under transferable power hypothesis. This hypothesis means that in the uplink, the sum-power is constrained but not the individual powers. This MAC with transferable power represents an optimistic model, but guarantees that BC bounds constitute outer bounds for the MAC, while providing a more tractable expression.

FBL information theory has been initially extended to MAC and BC scenarios in [14]. Gaussian MAC has been particularly investigated in [15,16] among others, and achievable rate regions have been characterized. Interesting results can also be reported on the dispersion of Gaussian BC in [17]. Unsal et al. have also investigated the Gaussian BC dispersion with superposition coding (SC) [18] leading to an achievable bound. However, several issues may limit the applicability of these bounds to the IoT context starting by the decoding error probability definition, which is often a joint probability and thus not suitable for a massive connectivity in an IoT context. Moreover, the achievability bound defined with joint-rate region also limits the insights that an IoT operator may extract from these expressions, and existing results are often limited in the number of users considered.

Fundamental bounds with many MAC users have recently been investigated from complementary perspectives. The authors in [19] gave bounds on the joint decoding error probability and capacity region when the number of users grows exponentially or sub-exponentially fast with respect to the number of c.u. and when their communications are asynchronous. The asynchronism and the number of users are linked exponentially with the number of c.u. The main conclusion is that reliable transmission (i.e., vanishing error probability) is impossible when the asynchronism is much more important than the number of users, but it remains possible when the number of users is sub-exponential with respect to the number of c.u. However, the authors focused on joint decoding error probability and typicality-based decoders. The authors of [20] studied a similar problem to that in [19] but when the number of users *K* grows linearly with the number of c.u. *n*, i.e., K=μn where μ is the user density. Moreover, the authors considered the per-user decoding error probability criterion, which is a much more relevant metric than the joint decoding error probability, when the number of users is large. The authors gave achievable and converse bounds on the minimal energy per bit for which reliable communication is possible; i.e., vanishing error probability, in the many-user MAC. However, they did not consider second-order expansions as introduced by [8] for Gaussian channels. In [21] the authors defined the many-access channel, which considers a large number of users in the MAC, and they studied the performance when the number of users grows. This work has been further extended in [22] and provided a fundamental limit for the sum-rate. However this model is not connected to the radio cell physical parameters. In [23], the authors explored the fundamental limit of the massive access, taking into account random packet arrivals and decoding error probabilities. The model is quite realistic and complementary to our work because the finite block-length regime is not considered, and specific random access policies are evaluated. The impact of random policies has also been investigated in [24], where the information aging is controlled.

Compared to these contributions, our work introduces the use of the spatial continuum broadcast and multiple access Gaussian channels (SCBC and SCMAC) [25] to model a spatial density of users and physical channel parameters, associated with the finite block-length analysis, to introduce latency and decoding error probability constraints. This model allows one to obtain an achievable bound for the symmetric rate case in the finite block-length regime.

### 1.2. Contributions and Related Work

In the context of IoT, the dense deployment of a large number of nodes in a finite area implies reconsidering the BC/MAC with a spatial distribution of the nodes leading to SCBC/SCMAC models [25,26], well adapted to represent NOMA cellular systems. This new model provided the fundamental EE-SE trade-off under equal-rate conditions in the asymptotic regime. This trade-off can be interpreted as an equivalent of the asymptotic Shannon capacity for a wireless cell with an ultra-dense distribution of users, when every user requests the same rate. The minimal power requested to satisfy a continuum of users using NOMA has been derived with SC in the asymptotic regime.

While these results provide interesting insights, on the maximal load of dense cells, latency and reliability were kept off the study. Hence, the critical question for IoT networks relates to estimating the price of latency and reliability constraints. As we shall see using FBL formalism, latency and reliability constraints come essentially at the cost of a reduction in the EE-SE region.

To measure this cost, two complementary issues are investigated in this paper. Firstly, to avoid the infinite time transmission induced by the asymptotic regime, a finite time constraint is introduced in the model, following [27] where the idea was introduced. The formal proof is provided with a discussion on its tightness based on simulation results. Secondly, transmission errors associated to transmitting small packets over finite time slots are modeled rigorously in the FBL regime. These two contributions allow to establish an achievable latency–reliability trade-off.

The core of our contribution lies in deriving the minimal requested power to serve a large number of users when the number of channel uses does not tend to infinity. That is, only a finite number of users can be superposed contrarily to [25,26]. This approach introduces a scheduling problem that can be reduced to a simpler splitting problem. Moreover, we show that for a given number of superposition levels, i.e., a finite number of splits of the cell, the scheduling order for two users belonging to the same level does not have any influence on the minimal requested power to serve the requested rate density.

The remaining of the paper is organized as follows. Section 2 introduces notations and the system model. Section 3 reviews our previous results on asymptotic SCBC. In Section 4, a finite time constraint (FTT) is introduced. Section 5 deals with decoding error probabilities associated with the FBL regime and estimates the impact on the achievable EE-SE trade-off. Finally, Section 6 draws conclusions and future works.

## 2. System Model

The model described below was first presented in [25].

### 2.1. Model and Parameters

A unique cell area denoted by $\Omega \subset R^{2}$ is served by a unique base station (BS). In this paper, a unique cell is considered without inter-cell interference. For a multi-cells extension, the reader can be referred to [28] where the inter-cell interference from a Poisson point process was considered, or to [29] where a fluid model was used. Further, the impact of cell geometry distribution has been explored in [30], but the association of the spatial continuum multi-cell and FBL is beyond the scope of this paper. (Ω,A,m) denotes the corresponding measurable space with A the Borel σ− algebra in Ω and *m* the Lebesgue measure. Without loss of generality, the BS is assumed to be located at point (0,0).

The measurable space (Ω,A,m) can be extended to $\left(\Omega \times T,A^{\prime },m^{\prime }\right)$ where $T=R^{+}$ represents the time and with $A^{\prime }$ the Borel σ− algebra in Ω×T and $m^{\prime }$ the associated Lebesgue measure.

Let U(x,t) be defined as a Poisson point process (PPP) on Ω×T, which represents the packet request arrivals at position *x* and time *t*. Thanks to the stationary properties of PPP, for any subset B∈A, the average number of user requests per time unit for one realization $\tilde{u}\left(x,t\right)$ is

(3) $$\lim_{T\to \infty }\frac{1}{T}\int_{t\in T}\int_{x\in B}\tilde{u}\left(x,t\right)\cdot dm^{\prime }\left(x,t\right)=U\left(B\right),$$

The global average number of requests per time unit associated with the whole cell service area Ω is denoted by $U_{T}$.

**Definition** **1** (Requested rate density)**.** *The requested rate density ρ:Ω→R is a Borel measurable function that represents the information rate spatial density ρ(x) requested at point x.*

The rate density is expressed in bit-per-channel-use (bpcu) per m^2^. A quantity measured in bpcu can be indeed converted into a physical rate for a real system using the number of channel uses per time unit, which relies on system parameters such as bandwidth, slots or frames. Clearly, a channel use can be interpreted as a resource element (RE) in an orthogonal frequency division multiple access system. However, its meaning is more general and may also correspond to one channel unit in any other access technology.

In addition, we are interested in this paper with the transmission of small packets. The first idea behind is the transmission of time-constrained small information quantities with no recurrent flows. This aspect is conventional in most papers related to the massive access for IoT. The second idea that relies on an information theory view considers that one packet is transmitted within a small number of channel uses (typically less than few hundreds). Under this assumption, the classical asymptotic regime used in information theory (e.g., [23,24,31]) does not hold, and the finite block-length regime needs to be used [19,20,21,22]. This constraint increases the difficulty of the mathematical analysis, but it also gives access to the latency versus reliability fundamental trade-off.

For the sake of simplicity, we further assume that all packets transport the same information quantity (in bits), denoted by $I_{0}$. This scenario is referred as the symmetric information, by analogy with the widely used property called symmetric rates in information theory. This assumption allows to keep the mathematical model tractable and is reasonable in many IoT applications.

Under this assumption the requested rate density of the cell relies directly on the user spatial density:

(4) $$\rho \left(x\right)=\frac{I_{0}u\left(x\right)}{N_{cu}},$$

where $N_{cu}$ is the number of channel uses per time unit and represents the bandwidth allocated to the system.

The cell sum-rate per channel use is called the spectral efficiency (SE) of the cell:

(5) $$\eta_{s}:=\int_{\Omega }\rho \left(x\right)dm\left(x\right).$$

For the symmetric information scenario, one has $\eta_{s}=\frac{I_{0}U_{T}}{N_{cu}}$.

In order to connect rate estimates to physical parameters of the cell, let be defined the equivalent noise, as the virtual noise level referenced back to the BS, where it matters for power allocation.

**Definition** **2** (Equivalent noise distribution)**.** *In a given radio cell in the downlink, for any receiver located at position x, the equivalent noise power is given by*

(6) $$\nu \left(x\right):=\sigma^{2}/g\left(x\right),$$

*where $\sigma^{2}$ is the receiver noise power, and g(x) is the channel power gain associated with this position.*

Without any fading nor shadowing, the maximal equivalent noise $\nu_{M}$ is obtained at the cell edge, while the minimal equivalent noise $\nu_{m}$ is obtained in the near field of the BS. Note that shadowing and fading are removed from the analysis for not to clutter the main output of the study, but the latter is general and can be easily extended considering fading and shadowing.

The requested rate density ρ(x) is distributed with respect to the equivalent noise associated with each request. Consider the following functions:

(7) $$\begin{array}{cc} G_{\nu }\left(\nu \right) & :=\frac{1}{\eta_{S}}\int_{\Omega }𝟙\left[\nu (x)\geq \nu \right]\rho \left(x\right)dm\left(x\right),\mathrm{and}\\ f_{\nu }\left(\nu \right) & :=-\frac{dG_{\nu }\left(\nu \right)}{d\nu }, \end{array}$$

where 𝟙[.] is the indicator function.

**Figure 1:**
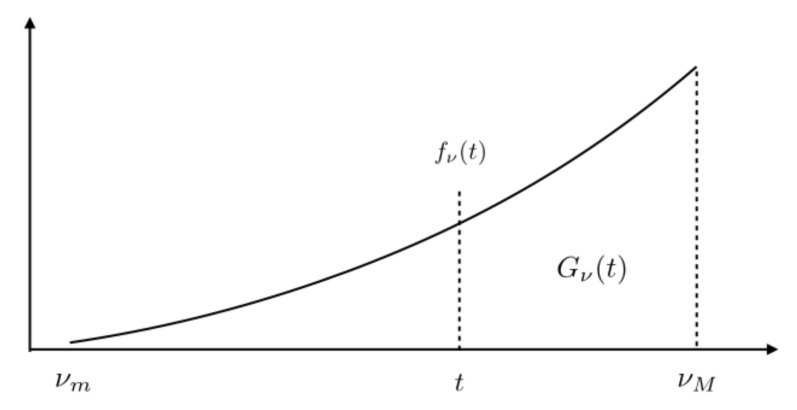
Rate request distribution $f_{\nu }\left(.\right)$ for a regular circular cell.

$G_{\nu }\left(\nu \right)$ represents the probability with which a packet request is made with an equivalent noise above ν (the most noisy requests). This is nothing but the complementary cumulative density function (ccdf) of the equivalent noise, with respect to the rate requests. Its derivation is therefore the probability density function (pdf) of ν with respect to the rate distribution. The meaning of $f_{\nu }\left(\nu \right)$ and $G_{\nu }\left(\nu \right)$ is illustrated in Figure 1 for a circular cell.

These definitions provide the key elements to characterize the set of rate distributions that are achievable under some power, latency, spectral efficiency and energy efficiency constraints.

### 2.2. Reference Scenario

Despite the fact that the model is general, the analytical results will be illustrated on a simplified reference scenario, for the sake of clarity, herein described.

The unique cell covers a disk of radius $R_{c}$ in the downlink mode. Simple power-law pathloss and omnidirectional antennas are considered with no shadowing. Hence, the channel gain is written as

(8) $$g\left(x\right)=g_{0}{\left|x\right|}^{-\alpha },$$

where $g_{0}$ and α represent, respectively, a reference pathloss and the attenuation slope. |x| is the geometric distance of the point *x* to the BS in (0,0). For numerical results, the following values are used: α=3.65 and $g_{0}=\sigma^{2}$. Additionally, the transmission power is constrained by a maximal power, i.e., $P\leq P_{M}$.

The rate demand is uniformly distributed, i.e., $\rho \left(x\right)=\rho_{0}$∀x∈Ω which relies on two assumptions: the symmetric rate hypothesis and a uniform spatial distribution of requests ($u\left(x\right)=u_{0}$). It follows that $\eta_{S}=m\left(\Omega \right)\rho_{0}=\pi R_{c}^{2}\rho_{0}$.

Under these assumptions, the equivalent noise distribution introduced in the former section is given by:

(9) $$\begin{array}{cc} f_{\nu }\left(\nu \right) & =\frac{2}{\alpha \nu_{c}}{\left(\frac{\nu }{\nu_{c}}\right)}^{2/\alpha -1},\;\forall \nu \in \left[0,\nu_{c}\right] \end{array}$$

(10) $$\begin{array}{cc} G_{\nu }\left(\nu \right) & =1-{\left(\frac{\nu }{\nu_{c}}\right)}^{2/\alpha },\;\forall \nu \in \left[0,\nu_{c}\right], \end{array}$$

with $\nu_{c}=\frac{\sigma^{2}}{g_{0}}R_{c}^{\alpha }$ the equivalent noise at the cell edge. The reader may refer to [25] for technical details.

### 2.3. Superposition Coding

SC is capacity achieving for Gaussian BC with successive interference cancellation (SIC) [32]. For a given set of $N_{u}$ users ordered according to their channel quality, i.e., from the strongest to the weakest, user 1 can only decode its own signal after having decoded the signals sent to users 2 to $N_{u}$, user 2 decodes its own signal after having decoded signals from users 3 to $N_{u}$ and so on. To make the rest of the paper clear, the main steps of SC are herein reviewed.

For two coded messages of length *n*, $X_{1}^{n}$ and $X_{2}^{n}$, assumed to be randomly drawn according to two independent distributions, i.e., $P_{X_{1}}^{n}$ and $P_{X_{2}}^{n}$, with average powers $P_{1}$ and $P_{2}$ respectively, the following holds. All decoding steps are done in an equivalent Gaussian channel, where $Z_{i}^{n}\left(k\right)∼N\left(0,\sigma^{2}I_{d}\right)$, ∀i∈{1,2}, and according to the following:

- The second user, with the largest equivalent noise, decodes its own signal in a Gaussian channel given by
  (11) $$Y_{2}^{n}=\sqrt{g_{2}}\cdot X_{2}^{n}+\left(\sqrt{g_{2}}\cdot X_{1}^{n}+Z_{2}^{n}\right).$$
  The normalized version of this equation is given by
  (12) $$Y_{2}^{{}^{\prime }n}=Y_{2}^{n}/\sqrt{g_{2}}=X_{2}^{n}+\left(X_{1}^{n}+\sqrt{\frac{1}{g_{2}}}\cdot Z_{2}^{n}\right).$$
  For this receiver the power of the equivalent additive Gaussian noise is $P_{1}+\nu_{2}$, and its maximum achievable rate in the asymptotic regime, i.e., n→∞, is $C\left(\frac{P_{2}}{P_{1}+\nu_{2}}\right)$.
- The first user, with the smallest equivalent noise, has two decoding iterations. It first has to decode the second user information in the following channel:
  (13) $$Y_{1}^{n}=\sqrt{g_{1}}\cdot X_{2}^{n}+\left(\sqrt{g_{1}}\cdot X_{1}^{n}+Z_{1}^{n}\right),$$
  with the normalized version given by
  (14) $$Y^{{}^{\prime }n}=X_{2}^{n}+\left(X_{1}^{n}+\sqrt{\frac{1}{g_{1}}}\cdot Z_{1}^{n}\right).$$
  For this receiver the power of the additive Gaussian noise is $P_{1}+\nu_{1}$, and the achievable data rate is $C\left(\frac{P_{2}}{P_{1}+\nu_{1}}\right)$. Then, after canceling the second user signal, receiver one decodes its own signal:
  (15) $$Y_{1}^{{}^{\prime \prime }n}=X_{1}^{n}+\sqrt{\frac{1}{g_{1}}}\cdot Z_{1}^{n},$$
  and can achieve its full achievable data rate, i.e., $C\left(\frac{P_{1}}{\nu_{1}}\right)$, in the asymptotic regime.

## 3. Symmetric Capacity in the Asymptotic Regime

### 3.1. Fundamental Trade-Off with SC

When SC is used, BS waits for a time *T* to aggregate a set of packet requests that are transmitted in the next slot to the corresponding nodes in *n* channel uses. Under no latency constraint, the time *T* can be taken arbitrarily large allowing to verify n→∞, corresponding to the asymptotic regime. The study of this regime leads to the access capacity region defined in [25] as the set of rate spatial distributions ρ(x) for which an encoder–decoder pair exists such that the transmission error tends to 0 when *T* tends to infinity.

**Figure 2:**
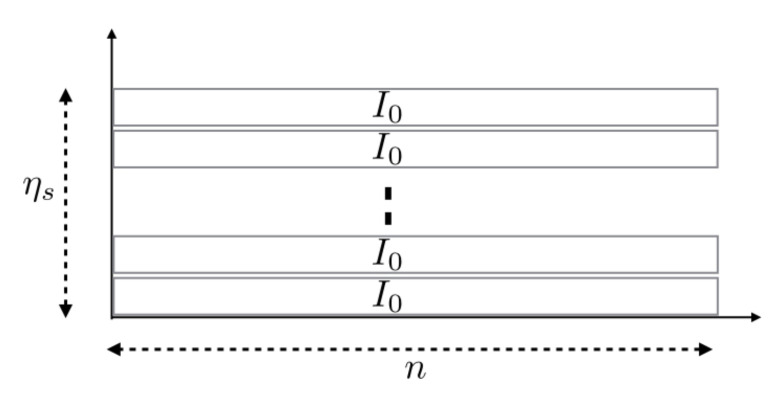
The asymptotic regime is obtained at the limit when n→∞. The cell spectral efficiency is kept constant, but the number of packets transmitted simultaneously tends to infinity. Each packet $I_{0}$ is spread over *n* channel uses, and the individual spectral efficiency tends to 0.

In comparison with Shannon’s asymptotic regime, our model adds a complementary parameter: when n→∞, the number of randomly distributed nodes (each node represents a message request) tends to infinity. Since we do not consider individual rates, but individual fixed information quantities $I_{0}$, the sum-rate converges to $U_{T}I_{0}$ while individual rates tend to 0 as they are equal to $I_{0}/n$. This is illustrated in Figure 2. It is worth noting that a cell’s sum-rate tends to its average spectral efficiency. In [25], based on an iterative splitting process, the maximal sum-rate the cell can achieve when a continuum of users is considered has been established. The corresponding fundamental limit is expressed as

**Theorem** **1** (GSCBC fundamental limit [25]). *The achievable EE-SE trade-off for a given rate spatial density ρ(x) is given by*

(16) $$\eta_{E}\leq {\left[a\int_{\nu_{m}}^{\nu_{M}}tf_{\nu }\left(t\right)\cdot e^{a\eta_{S}G_{\nu }\left(t\right)}dt\right]}^{-1},$$

*where a=2log(2) and $\eta_{E}$ is the energy efficiency.*

This result can be applied to the reference scenario described in Section 2.2, using Equation (9) in Equation (16), which yields

(17) $${\tilde{P}}_{m}=\nu_{M}\frac{a\eta_{S}}{1+\alpha /2}{}_{1}\;F_{1}\left(1;2+\alpha /2;a\eta_{S}\right),$$

with ${}_{1}\;F_{1}\left(a;b;x\right)$ the confluent hypergeometric function ([33], Section 9.21). ${\tilde{P}}_{m}$ is the minimal transmission power required at BS to serve the rate spatial density ρ(x).

The fundamental EE-SE limit of the corresponding cell is provided by Equation (17). Given the power normalized by the equivalent noise at the cell edge $p_{r}={\tilde{P}}_{m}/\nu_{c}$, EE in bppu is defined as $\eta_{E}=\eta_{S}/p_{r}$, leading to the fundamental EE-SE limit

(18) $$\eta_{E}=\frac{1+\alpha /2}{a\cdot {}_{1}\;F_{1}\left(1;2+\alpha /2;a\eta_{S}\right)}.$$

The EE should be understood as the total number of bits the base station can transmit under a transmission power constraint expressed as the relative sum-power received by an edge user. So, the term $1/\eta_{E}$ plays for the symmetric SCBC the role of the classical $E_{b}/N_{0}$ for a point-to-point link. Clearly, Equation (18) is equivalent to the Shannon’s second theorem in Equation (1), for the symmetric SCBC. The symmetric SCBC capacity C(γ) is obtained by inverting Equation (17) with respect to $\eta_{S}$ and denoting by $\gamma =\frac{{\tilde{P}}_{m}}{\nu_{M}}$ the SNR at the cell edge.

### 3.2. Fundamental Trade-Off with Orthogonal Sharing

A classical alternative to SC is to exploit orthogonal multiple access (OMA), e.g., by time division. In this case, to maximize the symmetric information, the BS allocates a fixed number of channel uses to each packet, and the transmission power adapts to constantly preserve the spectral efficiency. The transmission power used for a node in *x* is

(19) $$\frac{1}{a}\log\left(1+\frac{P(x)}{\nu (x)}\right)=\eta_{s}$$

**Lemma** **1** (Achievable bounds with OMA)**.** *In a single cell under the spatial continuum model, the fundamental EE-SE trade-off achievable by orthogonal multiple access is*

(20) $$\eta_{E}=\frac{1}{2}\frac{2+\alpha }{e^{a\eta_{S}}-1}\eta_{S}.$$

**Proof.**  See Appendix A. □

The corresponding curve is given as a baseline in Figure 4 where the EE-SE limit is represented. The blue curve represents the EE-SE fundamental limit achievable with OMA with α=3.65, and the red curve the fundamental limit established with NOMA (SC), plotted using Theorem 1.

To sum up, this section reported the fundamental EE-SE limit of the SCBC in the asymptotic regime as derived in [25]. Note that the *asymptotic regime* refers to a doubly asymptotic regime. Indeed, when n→∞, it follows that ϵ→0 but with the SCBC, the number of nodes transmitting simultaneously also tends to infinity with individual rates going to 0. However, the sum-rate converges to the SCBC capacity.

## 4. Finite Time Transmission Constrained Model

The objective of this paper is to introduce a transmission time constraint into the former model to obtain achievable bounds of NOMA under more realistic assumptions than the doubly asymptotic regime.

Consider the situation in which each packet has to be transmitted in a finite time T∈N, i.e., within a finite number of channel uses. For the moment, we still consider arbitrary low error probabilities, sustained with γ and *n* sufficiently high. This hypothesis will be relaxed in Section 5.

### 4.1. FTT Formulation

This constraint can be formalized as follows:

**Definition** **3** (Finite Time Transmission Constraint)**.** *A multi-user network with packets of $I_{0}$ bits, with $I_{0}\in N$, is said to be FTT constrained when each transmission lasts at most $n^{*}$ channel uses.*

This definition only imposes that a packet of $I_{0}$ bits is transmitted in at most $n^{*}$ channel uses, but the queuing delay is not controlled. The FTT constraint is then a necessary but not sufficient condition for delay-constrained transmissions. The FTT constraint provides interesting insights anyway. For instance, it allows to setup the transmission duration of each packet, thereby controlling the activity time of each receiver.

To assess the symmetric rate fundamental limit of the cell under the FTT constraint, let us review that the average spectral efficiency (i.e., the sum-rate) of the cell noted ${\bar{\eta }}_{s}$ shall be equal to

(21) $${\bar{\eta }}_{s}=\frac{I_{0}U_{T}}{N_{cu}}.$$

When the BS transmits a packet of $I_{0}$ bits in $n^{*}$ channel uses, the individual rate for this packet is $\eta_{u}=\frac{I_{0}}{n^{*}}$. In order to achieve the target spectral efficiency ${\bar{\eta }}_{s}$, the BS has to use SC to transmit simultaneously several packets. Therefore, the FTT constrained problem is equivalent to the following scheduling problem:

**Definition** **4** (SC Scheduling policy)**.** *The following are given:*

- a frame of $N_{cu}$ channel uses of duration T, itself divided into L slots, each slot $s_{l};\forall l\in \left\{1,2,\cdots ,L\right\}$ contains $n^{*}$ channel uses. One has $N_{cu}=Ln^{*}$;
- a BS’s queue containing a random number of packets to be transmitted to a set of nodes $N_{U}$, selected according to the PPP U(x,t) restricted to the subset Ω×T.

*A SC scheduling policy selects a subset of users $N_{u}\left(l\right)\subset N_{U}$ for each slot $s_{l}$, which are ordered with their increasing equivalent noise, i.e., $\nu_{k+1}\left(l\right)>\nu_{k}\left(l\right)$. Decoding is performed at each user, according to the SC technique.*

The number of users associated to each slot $s_{l}$ is noted $N_{u}\left(l\right)=\left|N_{u}\left(l\right)\right|$, and the corresponding spectral efficiency is

(22) $$\eta_{S}\left(l\right)=N_{u}\left(l\right)\eta_{u}.$$

### 4.2. Optimal Scheduling Policy

We now propose to determine an optimal scheduling policy in the asymptotic regime.

**Definition** **5** (Optimal scheduling policy)**.** *A scheduling policy for the PPP U(x,t) over Ω×T is asymptotically optimal under a FTT constraint, if all user requests are served within $n^{*}$ channel uses at most, and if the transmission power is minimal over all possible scheduling policies, when T→∞.*

Note this asymptotic regime is conditioned on the FTT constraint and is, thus, more constrained than the regime studied in Section 3.

Let $\gamma_{k}\left(l\right)$ be the effective signal-to-interference-plus-noise-ratio (SINR) for node $u_{k}\left(l\right)$, defined as

(23) $$\gamma_{k}\left(l\right)=\frac{P_{k}\left(l\right)}{\nu_{k}\left(l\right)+\sum_{i<k}P_{i}\left(l\right)}.$$

This SINR is effective when the appropriate decoding is used and thanks to the superposition coding principle.

Since each user needs to get a reliable individual rate $\eta_{u}=I_{0}/n^{*}$, its effective SINR $\gamma_{k}\left(l\right)$ needs to verify

(24) $$\gamma_{k}\left(l\right)\geq \gamma^{*},\forall \left(k,l\right),$$

where l∈{1,2,⋯,L} and $k\in \{1,2,\cdots ,N_{u}\left(l\right)\}$ and with $\gamma^{*}=e^{a\eta_{u}}-1$, according to the channel capacity theorem.

It is then straightforward to say that the optimal power used for each packet in slot $s_{l}$ is given by

(25) $$\begin{array}{cc} P_{1}\left(l\right) & =\gamma^{*}\cdot \nu_{1}\left(l\right),\\ P_{2}\left(l\right) & =\gamma^{*}\cdot \left(\nu_{2}\left(l\right)+P_{1}\left(l\right)\right),\\ \; & \vdots \\ P_{N_{u}\left(l\right)} & =\gamma^{*}\cdot \left(\nu_{N_{u}}\left(l\right)+P_{1}\left(l\right)+\cdots +P_{N_{u,l}-1}\left(l\right)\right). \end{array}$$

The BS transmission power for slot *l* is then $P_{m}\left(l\right)=\sum_{k}P_{k}\left(l\right)$.

Then, in this symmetric rate setup, where all nodes require the same SINR, the following Lemma holds, where the slot numbering is omitted for the sake of clarity.

**Lemma** **2** (Minimum sum-power in the symmetric Gaussian BC)**.** *Given a set of users indexed by $k\in \{1,\cdots ,N_{u}\}$, ordered such that $\forall k,\nu_{k}\geq \nu_{k-1}$, the minimum sum-power necessary to transmit reliably to each node independent information $I_{0}$, under effective SINR $\gamma^{*}$, is*

(26) $$P_{m}=\sum_{\bar{k}=1}^{N_{u}}d_{\bar{k}}\cdot \nu_{\bar{k}},$$

*with $d_{\bar{k}}:\;=\gamma^{*}{\left(1+\gamma^{*}\right)}^{\bar{k}-1}$, and $\bar{k}=N_{u}-k+1$.*

**Proof.**  The proof relies on the decomposition of $P_{k}$ according to Equation (25), i.e., $P_{k}=\sum_{j=1}^{k}c\left(k,j\right)\cdot \nu_{j}$, where the c(k,j), represented in Table 1, are given by

(27) $$\begin{array}{cc} \; & c(k,j)=0;\;\forall j>k,\\ \; & c\left(k,k\right)=\gamma^{*},\\ \; & c\left(k,j\right)=\gamma^{*}\sum_{i=j}^{k-1}c\left(i,j\right);\;\forall j<k. \end{array}$$

These coefficients c(k,j), can be computed recursively, with $c\left(k,k\right)=\gamma^{*}$ and $c\left(k,k-1\right)=\gamma^{*2}$, and the following recursion for j<k−1:

(28) $$\begin{array}{cc} c(k,j) & =\gamma^{*}\sum_{i=j}^{k-1}c\left(i,j\right),\\ \; & =\gamma^{*2}{\left(1+\gamma^{*}\right)}^{k-j-1}. \end{array}$$

In Table 1, each row represents a decomposition of the power of one message, with respect to the equivalent noise ν(k) of all users. In parallel, the sum-power can be computed column-wise first, leading to

(29) $$P_{m}=\sum_{k=1}^{N_{u}}d\left(k\right)\cdot \nu_{k},$$

with $d\left(k\right)=\sum_{i=k}^{N_{u}}c\left(i,k\right)$. Using Equation (27), these coefficients can be straightforwardly rewritten as

(30) $$\begin{array}{cc} d(k)\; & =\gamma^{*-1}\cdot c\left(N_{u}+1,k\right). \end{array}$$

Then, with Equation (28), one obtains $d\left(k\right)=\gamma^{*}{\left(1+\gamma^{*}\right)}^{N_{u}-k}$ leading to the final expression:

(31) $$P_{m}=\gamma^{*}\sum_{k=1}^{N_{u}}\nu_{k}\cdot {\left(1+\gamma^{*}\right)}^{N_{u}-k}.$$

Now, numbering the nodes in the reverse order (noted $\bar{k}$ for clarity), i.e., from the farthest to the nearest one, ends the proof. □

This theorem shows that $P_{m}$ is a linear combination of the equivalent noises $\nu_{\bar{k}}$ weighted with $d_{\bar{k}}$. Then, each coefficient relies only on $\gamma^{*}$ and grows exponentially with $\bar{k}$. The optimal strategy, which minimizes $P_{m}$, should obviously allocate the users according to their channel quality.

We draw the reader’s attention to the fact that the $\bar{k}$-th term of the sum in Equation (26) should not be interpreted as the power used to transmit the $\bar{k}$-th message but as the additional power induced by the $\bar{k}$-th equivalent noise level in the sum-power. The power associated to each message is given by (25). Nevertheless, the linear relation in Equation (26) of Lemma 2 is more appropriate to demonstrate the optimality of the proposed scheduling policy.

Consider a set of *L* slots and a set of users $N_{U}$ requesting a message, with $N_{U}=\left|N_{U}\right|$. A scheduling policy associates $N_{u}\left(l\right)$ users for each slot $s_{l}$. Let us recall that, according to our definition, the users $u_{1}\left(l\right)\cdots u_{N_{u}\left(l\right)}\left(l\right)$ are ordered with respect to their equivalent noise. Using the notation of Lemma 2, we refer to $\bar{k}$ as the coding level. A message encoded at level $\bar{k}$ means that the corresponding receiver needs to decode first the packets of lower level.

**Definition** **6** (Natural ordering policy)**.** *Assume that $N_{u}\left(l\right)=N_{u}$, ∀l, and then $N_{U}=L\times N_{u}$. This comes without loss of generality, as shown at the end.*

*Let now the nodes in $N_{U}$ be ordered from the strongest to the weakest equivalent noise, from $u_{1}$ to $u_{N_{U}}$. The natural ordering policy proceeds by assigning the users $u_{1}$ to $u_{L}$ to the first coding level over the L slots. Once the first coding level is filled out, the second level is filled and so on up to the last coding level. It follows that*

(32) $$u_{\bar{k}}\left(l\right)=u_{l+(k-1)L},$$

This scheduling policy is illustrated in Table 2.

**Theorem** **2** (Optimal scheduling)**.** *For a given set of users indexed by $\left\{1,\cdots ,N_{U}\right\}$ and ordered from the strongest to the weakest equivalent noise, the natural ordering policy is optimal with respect to the average transmission power.*

**Proof.**  From Lemma 2, it follows that the natural ordering must be used. The remaining question is about the repartition of the users through the different slots.

To prove that the natural ordering policy is optimal, let us consider another policy, for which one of the first *L* users noted $u_{i}$ is not allocated to the first coding level, but to the level ${\bar{k}}_{i}$. Then, there exists a user $u_{j}$ allocated to the first coding level, such that j>L. Then, a simple permutation noted $\pi (u_{i},u_{j})$ is sufficient to reduce the sum power, since the equivalent noise of $u_{j}$ is lower than that of $u_{i}$, and the power difference between the two policies is

(33) $$\Delta P=\left(d_{{\bar{k}}_{j}}-d_{{\bar{k}}_{i}}\right)\left(\nu_{i}-\nu_{j}\right),$$

which is strictly negative.

So starting from any policy, moving all the *L* first users with permutations to the first coding level reduces the sum-power. Then, proceeding the same way as the higher-order coding levels will also reduce the power. At the end of these permutations, each coding level $\bar{k}$ contains the same users as the natural ordering policy.

It should be also noted that any permutation between two users at the same coding level does not change the sum power. Therefore, the natural ordering policy is one of the optimal ones. It is worth noting that when doing such a permutation, the individual power allocated to each message may change, but with no impact on the sum-power. This completes the proof. □

Finally, if $N_{U}=L\times N_{u}$ is not verified, the same permutations can be used, and one obtains a policy where the last coding level is partially filled in. In this case, the number of levels (or superposition codes) is given by $\left\lceil \eta_{S}/\eta_{u}\right\rceil$.

### 4.3. Optimal Scheduler When T→∞

According to the previous result, the optimal scheduler relies on transmitting at each round to exactly $N_{u}$ users with $N_{u}=\frac{\eta_{s}}{\eta_{u}}$. Let us assume this ratio to be an integer. If it is not the case, alternate rounds may be used with $\left\lfloor \frac{\eta_{s}}{\eta_{u}}\right\rfloor$ and $\lfloor \frac{\eta_{s}}{\eta_{u}}\rfloor +1$ users.

**Figure 3:**
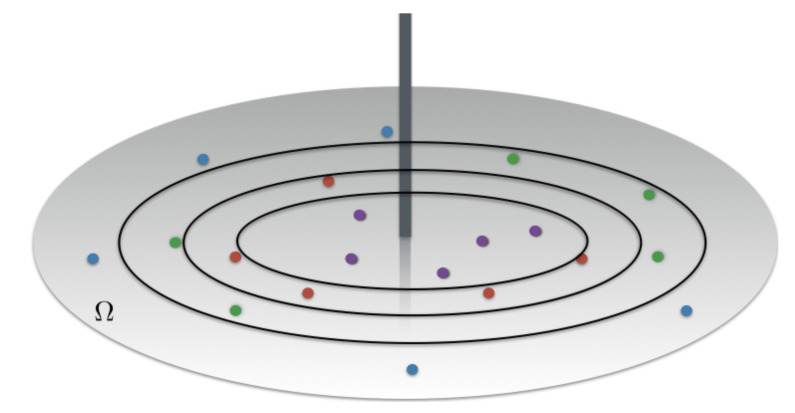
The optimal scheduling requires to divide the cell in equal rate subsets as a function of their channel quality.

The optimal scheduling policy is enforced by partitioning the cells in $N_{u}$ subsets of equivalent sum-rates:

(34) $$\tilde{B}:=\left\{B_{1},\cdots B_{N_{u}}\right\},$$

where $B_{\bar{k}}:=\left\{x;\nu \left(x\right)\in \left[\nu_{\bar{k}};\nu_{\bar{k}-1}\right)\right\}$, as illustrated in Figure 3 for a regular circular cell. The thresholds $\nu_{k}$ are defined with $\nu_{0}=\nu_{M}$, $\nu_{N_{u}}=\nu_{m}$, and such that $|B_{\bar{k}}|=U_{T}/N_{u};\forall k$ with $U_{T}$ the total number of requests. Once this partition is done, at each slot, the BS picks up a user per subset and transmits to these users with SC.

This scheduler achieves the minimal average power when *T* and *L* tend to infinity. Indeed, the partition $B^{\left(\infty \right)}$ converges to a partition where all $B_{\bar{k}}$ are of equal surface (due to the properties of the uniform PPP model), and the asymptotic average power is given by

(35) $${\bar{P}}_{m}=\lim_{L\to \infty }\frac{1}{L}\sum_{l=1}^{L}\sum_{\bar{k}=1}^{N_{u}}d_{\bar{k}}\cdot \nu_{\bar{k},l},$$

which can be expanded as

(36) $$\begin{array}{cc} {\bar{P}}_{m}\; & =\sum_{\bar{k}=1}^{N_{u}}d_{\bar{k}}\cdot E_{x\in B_{\bar{k}}^{\left(\infty \right)}}\left[\nu \left(x\right)\right],\\ \; & =\sum_{\bar{k}=1}^{N_{u}}d_{\bar{k}}\cdot E_{\nu \in [\nu_{\bar{k}};\nu_{\bar{k}-1})}\left[\nu \right]=\sum_{\bar{k}=1}^{N_{u}}d_{\bar{k}}\cdot {\bar{\nu }}_{\bar{k}}, \end{array}$$

where ${\bar{\nu }}_{k}$ stands for the average equivalent noise over the $\bar{k}$-th subset.

Using the expression of $d_{\bar{k}}$ and expanding $\gamma^{*}$, one obtains

(37) $$\begin{array}{ccc} {\bar{P}}_{m} & = & \left(e^{a\eta_{u}}-1\right)\cdot \sum_{\bar{k}=1}^{N_{u}}e^{a\eta_{u}\left(\bar{k}-1\right)}{\bar{\nu }}_{\bar{k}}, \end{array}$$

(38) $$\begin{array}{ccc} & \approx & a\eta_{s}\cdot \sum_{\bar{k}=1}^{N_{u}}\frac{e^{a\eta_{u}\left(\bar{k}-1\right)}{\bar{\nu }}_{\bar{k}}}{N_{u}}. \end{array}$$

The last approximation comes when the number of subsets is sufficiently large such that $a\eta_{u}<<1$.

Interestingly, this result can be compared to the fundamental limit established in Theorem 1. These expressions are similar, except that the continuous integral has been replaced by a discrete sum, and the equivalent noise ν by ${\bar{\nu }}_{\bar{k}}$. The term $a\eta_{s}G_{\nu }\left(t\right)$ in the exponential is replaced by its discrete version $a\eta_{u}k$ and $f_{\nu }\left(t\right)$ by $1/N_{u}$. It is then straightforward to show that Theorem 1 is obtained as the limit of Equation (38) when $N_{u}$ tends to infinity, i.e., when the constraint $n^{*}\to \infty$, which proves the doubly asymptotic optimality of this scheduler.

### 4.4. Application Example

**Figure 4:**
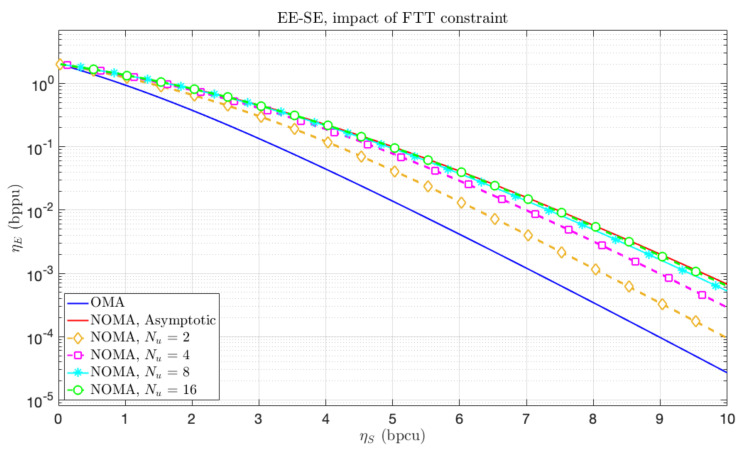
$\eta_{E}-\eta_{S}$ trade-off for the baseline OMA (blue), the asymptotic NOMA (red) and for FTT models with $N_{u}\in \left\{2,4,8,16\right\}$.

The former analytical results are applied to the reference scenario of Section 2.2 and represented in Figure 4 with the cross curves for different numbers of SC layers (indicated by $N_{u}$). Moreover, the path loss exponent is α=3.65, and the reference path loss and the noise power are normalized, i.e., $g_{0}=\sigma^{2}=1$. The two asymptotic curves are given in blue for OMA, i.e., with Lemma 1 and red for NOMA, i.e., with Theorem 1. The orange curve with diamonds corresponds to the 2-user NOMA. The green, cyan and magenta curves with square, stars or circles are obtained with 4, 8 and 16-user NOMA, respectively. These curves are obtained by applying Equation (37), where $\eta_{u}$ is simply the total spectral efficiency $\eta_{S}$ divided by the number of users, since all users receive the same amount of information. These curves highlight the performance loss when the number of superposed codes is equal or lower than 4. This model also shows that the fundamental limit established for NOMA in the asymptotic regime is almost achievable with a reasonable number of coding levels; 90% of the gain is achieved with only 4 coding levels, and even 30% of the gain is achieved with 2 coding levels.

**Figure 5:**
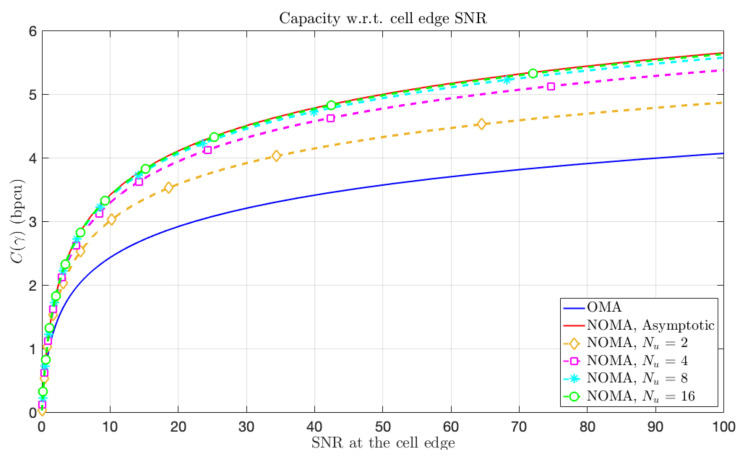
NOMA Cell capacity C(γ) under symmetric-information for a given SNR γ at cell edge (red), achievable symmetric-rate with OMA (blue) and capacity under FTT constraint with $N_{u}$ layers for $N_{u}\in \left\{2,4,8,16\right\}$.

In addition, the capacity of the cell is represented in Figure 5 in the same conditions. In both figures, one can remark the sub-optimality of OMA in the doubly asymptotic regime. Moreover, one can also observe the quick convergence to the optimal performance, i.e., the EE-SE Pareto front in Figure 4 and the asymptotic cell capacity in Figure 5, with the number of partitions of the cell.

Both figures highlight the interest of our fundamental limit given by Theorem 1, being almost achievable with a NOMA strategy.

## 5. Finite Block-Length (FBL) Constrained Model

The last step addressed in this section to compare practical NOMA schemes to the fundamental limit is to relax the error-free assumption to cope with the FBL regime, more appropriate for small packets.

We herein develop an approximation of the achievability bound with a NOMA scheme (SC) by exploiting the normal approximation derived in [8] for a point-to-point transmission and reviewed in Equation (2).

For a fixed number of bits $I_{0}$ to be transmitted, Equation (2) leads to

(39) $$I_{0}\approx nC\left(\gamma \right)-Q^{-1}\left(\epsilon \right)\cdot \sqrt{n\cdot V(\gamma )},$$

which provides a relationship between $P_{m}$ (through γ), *n* and ϵ. Consider a 2-users BC before the generalization to $N_{u}$-users BC. In the following, we denote $\epsilon_{i,j}$ the decoding error probability of message *j* by user *i*.

### 5.1. Achievable Minimal Power for the 2-User Gaussian BC

Let us review that in our setup, the BS aims at transmitting two independent packets of $I_{0}$ bits each, to two users in Gaussian channels in at most *n* channel uses and with an average individual error probability lower than $\epsilon^{*}$ for each user, i.e., $\epsilon_{i}\leq \epsilon^{*}$, $\forall i\in \{1,\cdots ,N_{u}\}$.

Considering the targeted rate is $R^{*}=I_{0}/n$ and assuming the interference caused by the other users to be Gaussian, we can write for the weakest user:

(40) $$C\left(\gamma_{2}^{*}\right)-Q^{-1}\left(\epsilon^{*}\right)\sqrt{\frac{V(\gamma_{2}^{*})}{n}}\geq \frac{I_{0}}{n}.$$

Contrary to the asymptotic situation described in Section 4, the target SNR value needs to be adapted for each user as a consequence of the SC technique.

Considering user 2, $\gamma_{2}^{*}$ is obtained as the unique solution of Equation (40) for some tuple $\left(n,\epsilon^{*},I_{0}\right)$. The solution is unique because this equation is monotonically increasing with respect to γ. This imposes the following relation between $P_{1}$ and $P_{2}$:

(41) $$P_{2}\geq \gamma_{2}^{*}\left(P_{1}+\nu_{2}\right).$$

Now considering user 1, it first decodes message 2 with a lower error noted $\epsilon_{1,2}<\epsilon^{*}$, because the SNR is stronger.

By the union bound, the decoding error probability of user 1 is bounded by the sum of the decoding errors associated with the two messages $\epsilon_{1}\leq \epsilon_{1,1}+\epsilon_{1,2}$. Then, to keep a global error probability lower than $\epsilon^{*}$, the error probability on its intended message $\epsilon_{1,1}$, should satisfy

(42) $$\epsilon_{1,1}\leq \epsilon^{*}-\epsilon_{1,2}.$$

Therefore, the minimum required SNR for the strongest user, $\gamma_{1}^{*}$, is the solution of

(43) $$C\left(\gamma_{1}^{*}\right)-Q^{-1}\left(\epsilon^{*}-\epsilon_{1,2}\right)\sqrt{\frac{V(\gamma_{1}^{*})}{n}}=\frac{I_{0}}{n},$$

which is bigger than $\gamma_{2}^{*}$ because the error constraint is stronger.

Solving these equations provides the minimal transmission powers $P_{1}$ and $P_{2}$ as

(44) $$\begin{array}{cc} \; & P_{1}=\gamma_{1}^{*}\cdot \nu_{1},\mathrm{and}\\ \; & P_{2}=\gamma_{2}^{*}\cdot \left(P_{1}+\nu_{2}\right)=\gamma_{2}^{*}\cdot \left(\gamma_{1}^{*}\cdot \nu_{1}+\nu_{2}\right). \end{array}$$

Although an analytic expression cannot be written, numerical computation is straightforward.

### 5.2. Impact of the Power Sharing between $P_{1}$ and $P_{2}$

**Figure 6:**
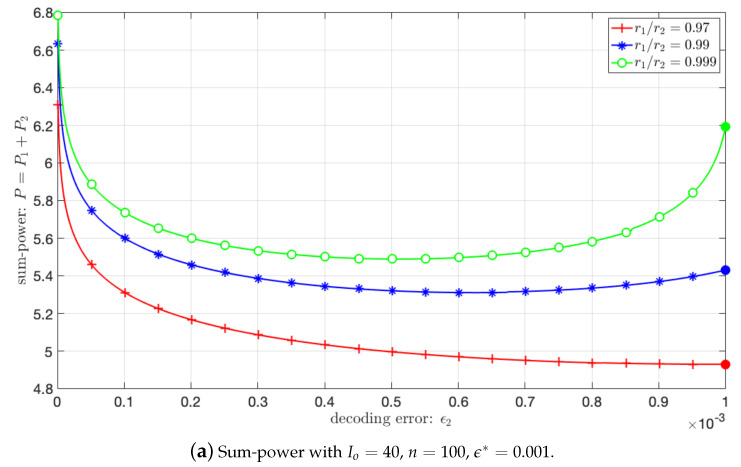

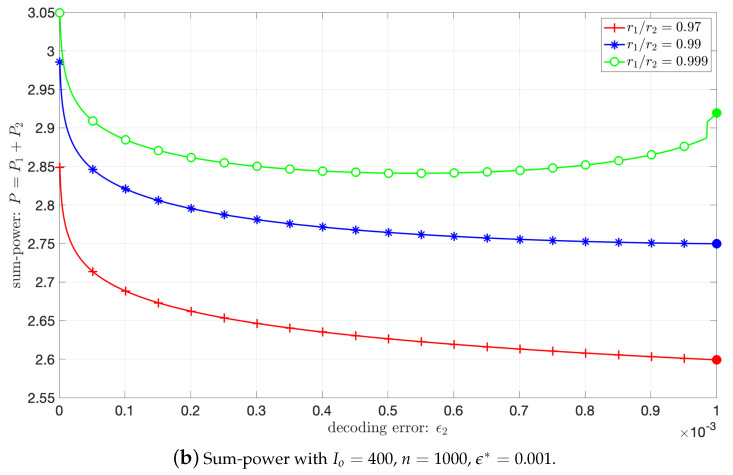
Sum-power with $I_{o}=400$, n=1000, $\epsilon^{*}=0.001$.

In Equation (40), we determined the minimal power allowing to achieve the error target on user 2. However, the use of a larger power $P_{2}$ could be justified from a theoretical point of view, since it would allow to reduce $\epsilon_{1,2}$, then allowing to reduce $P_{1}$ as the solution to Equation (43). The influence of reducing $\epsilon_{2}$ on the sum-power is illustrated in Figure 6 for the simulation parameters described in Section 2.2 and for a target individual error probability $\epsilon^{*}=10^{-3}$. The sum-power is plotted for different information size and block-lengths ($\left(I_{0}=40,n=100\right)$, and $\left(I_{0}=400,n=1000\right)$). Each curve is obtained when the users are positioned at distance $r_{1}$ and $r_{2}$ from the BS.

The reference solution obtained with $\epsilon_{2}=\epsilon^{*}$ is on the right of each plot (indicated with a plain circle). A sum-power reduction by increasing $P_{2}$ and reducing $P_{1}$ exists but is significant only when $r_{1}/r_{2}$ approaches 1. Clearly, when SC is used for users with significantly different positions, the reference solution is nearly optimal. This is justified because when the SNRs of the two users are sufficiently different, then $\epsilon_{1,2}<<\epsilon_{2}=\epsilon^{*}$; therefore, the impact of $\epsilon_{1,2}$ in Equation (43) is negligible.

### 5.3. Achievable Power for the N-User BC

Extending the former result to the N-user Gaussian BC is straightforward with SC, when the power of each user is optimized according to Equation (43). At each level, an additional penalty on the error is introduced. $\gamma_{\bar{k}}^{*}$ is thus the solution to

(45) $$C\left(\gamma_{\bar{k}}^{*}\right)-Q^{-1}\left(\epsilon^{*}-\sum_{j=1}^{\bar{k}-1}\epsilon_{\bar{k},j}\right)\sqrt{\frac{V(\gamma_{\bar{k}}^{*})}{n}}=\frac{I_{0}}{n}.$$

The sum introduced in the $Q^{-1}$ function shows how the error probabilities accumulate, which is the key issue of a SC approach in FBL regime.

The consequence for the sum-power follows from the iterative relations:

(46) $$P_{\bar{k}}\geq \gamma_{\bar{k}}^{*}\cdot \left(\nu_{\bar{k}}+\sum_{j=\bar{k}+1}^{N_{u}}P_{j}\right),$$

where $\gamma_{\bar{k}}^{*}$ is the solution of Equation (45).

**Figure 7:**
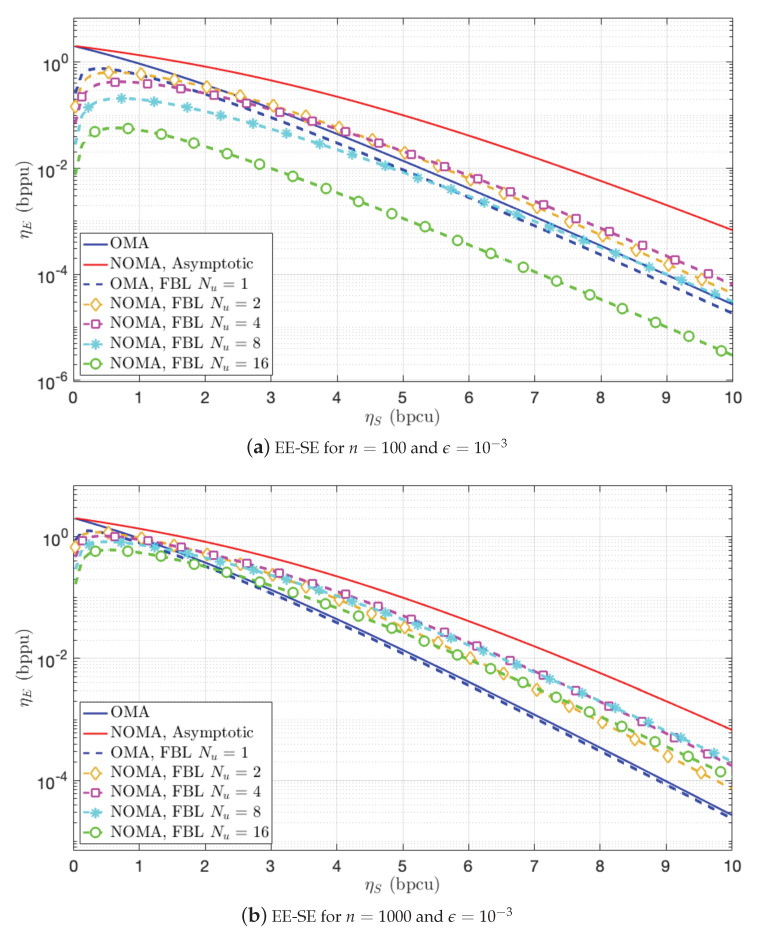
EE-SE trade-off for different number of channel uses *n* and for a per-user probability of error $\epsilon =10^{-3}$.

The NOMA achievable EE-SE trade-off for two different block-lengths (n=100 or n=1000) and an individual error probability threshold $\epsilon^{*}=10^{-3}$ is represented in Figure 7 with the iterative power allocation described above for a number of coding levels in $N_{u}\in \left\{1,2,4,8,16\right\}$. The EE-SE trade-off of OMA in asymptotic and FBL regimes is also plotted for reference.

Clearly, for small block-length (n=100, Figure 7a), the achievable region shrinks the most with the 16-user SC due to the impact of error accumulation. The best FBL SC configuration is the 4-user SC (green curve), in moderate to high spectral efficiency regime. The 2-user SC is almost optimal in these regimes. In the low spectral efficiency regime (below 1), OMA (dotted blue line) outperforms NOMA. When the block-length is larger (n=1000, Figure 7b), OMA remains optimal in the low spectral efficiency regime. However, in the moderate to high spectral efficiency regime, the degradation of SC reduces significantly, and all NOMA schemes outperform OMA. In this situation, the 4-user or 8-user SC are the best ones.

An important conclusion is that SC is inappropriate for very small packets at low SNR. This is in line with [20] that pointed out the better performance of OMA when the density of users μ=K/n<<1, with *K* the number of users, compared to a full decoder. Note that [20] considered a MAC scenario while we are considering BC in this paper. However, thanks to the MAC-BC duality, the conclusions could be easily transposed to the MAC scenario because, in both cases, successive decoding is used as a baseline.

## 6. Conclusions

In this paper, we proposed an analytic model to evaluate the performance of NOMA with many users when the transmission time is constrained and when small packets are transmitted. For that, we merged the spatial continuum model introduced in [25] with the finite block-length second-order rate expansion limit introduced by Polyanskiy et al.

We first show that the fundamental limit obtained with the spatial continuum model is relevant as this fundamental limit can be reached with a reasonable number of superposition coding layers when the messages are transmitted over large block-lengths. This result justifies the use of the proposed fundamental limit (Theorem 1) to optimize the design of cellular networks for NOMA IoT cells.

By exploiting a SC scheme in FBL, we further show the performance degradation when *n* reduces below 1000. However, it is worth mentioning that our FBL analysis relies on assumptions which prevent us from claiming that NOMA is necessarily worse than OMA in FBL. Indeed, (i) we used the normal approximation, (ii) we impose a SC strategy and (iii) we used a sub-optimal reference power allocation in SC.

Even if the normal approximation has been observed to be tight by simulation, the classical Berry–Esseen bounds are not sufficient to prove this tightness [8,16,17]. The recent paper [34] explores the tightness of saddle-point approximations for the P2P channel and could be used in the future to determine tighter bounds for the N-user BC. Nevertheless, additional simulations not presented here for the sake of consistency show that the degradation of SC in the FBL is not due to this approximation.

Concerning the SC strategy, clearly responsible of the performance degradation due to the successive decoding algorithm, an open question is to determine if a dirty paper coding technique in FBL could outperform the SC technique. Answering this question may rely on [17,35].

## Figures and Tables

**Table 1:** Power series coefficients c(k,j) (*k*th row, *j*th column) representing the coefficient of the *j*th equivalent noise into the *k*th power term.

	$\nu_{1}$	$\nu_{2}$	$\nu_{3}$	⋯	$\nu_{N_{u}}$
$P_{1}:$	$\gamma^{*}$			⋯	
$P_{2}:$	$\gamma^{*2}$	$\gamma^{*}$		⋯	
$P_{3}:$	$\gamma^{*3}+\gamma^{*2}$	$\gamma^{*2}$	$\gamma^{*}$	⋯	
$P_{4}:$	$\gamma^{*4}+2\gamma^{*3}+\gamma^{*2}$	$\gamma^{*3}+\gamma^{*2}$	$\gamma^{*2}$	⋯	
⋮					

**Table 2:** Optimal scheduling policy allocating the users through coding levels and slots.

		Slots				
		**1**	**2**	**3**	**⋯**	L
	$N_{u}$	$u_{N_{U}-L+1}$	$u_{N_{U}-L+2}$	$u_{N_{U}-L+3}$	⋯	$u_{N_{u}}$
$\bar{k}$	⋮	⋮	⋮	⋮	⋮	⋮
	2	$u_{L+1}$	$u_{L+2}$	$u_{L+3}$	⋯	$u_{2L}$
	1	$u_{1}$	$u_{2}$	$u_{3}$	⋯	$u_{L}$

## Abbreviations

The following abbreviations are used in this manuscript:

AWGN	Additive White Gaussian Noise
BC	Broadcast Channel
BS	Base Station
ccdf	complementary cumulative distribution function
EE	Energy Efficiency
FBL	Finite block-length
FTT	Finite Time Transmission (constraint)
GSCBC	Gaussian Spatial Continuum Broadcast Channel
IoT	Internet of Things
MAC	Multiple Access Channel
NOMA	Non-Orthogonal Multiple Access
OMA	Orthogonal Multiple Access
pdf	probability density function
PPP	Poisson Point Process
SC	Superposition Coding
SCBC	Spatial Continuum Broadcast Channel
SCMAC	Spatial Continuum Multiple Access Channel
SE	Spectral Efficiency
SIC	Successive Interference Cancellation
SINR	Signal-To-Interference-Plus-Noise Ratio
SNR	Signal-To-Noise Ratio
ULLC	Ultralow Latency Communications

## Appendix A. Proof of Lemma 1

Given Equation (19), the power needed at distance *r* is $P\left(r\right)=\left(e^{a\eta_{s}}-1\right)\frac{\sigma^{2}r^{\alpha }}{g_{0}}$. In the case of spatial continuum, the equi-repartition of the power among the users is the total power integrated over the surface divided over the area of the cell. In a circular cell, the power distributed over a ring located at distance *r* from the BS and whose the thickness is dr is P(r)·2πrdr. The power used in OMA is, hence, the total power spread over the cell divided by the total area. For a circular cell of radius $R_{c}$, we have

(A1) $$P_{\mathrm{OMA}}=\frac{\int_{\Omega }P\left(r\right)2\pi rdr}{\pi R_{c}^{2}}$$

After straightforward computation, we end up with

(A2) $$P_{\mathrm{OMA}}=\frac{2\sigma^{2}R_{c}^{\alpha }}{g_{0}\left(\alpha +2\right)}\left(e^{a\eta_{s}}-1\right)$$

EE is simply SE normalized by $P_{\mathrm{OMA}}/\nu_{c}$, and the proof is complete.
